# Calcium Flux across Plant Mitochondrial Membranes: Possible Molecular Players

**DOI:** 10.3389/fpls.2016.00354

**Published:** 2016-03-31

**Authors:** Luca Carraretto, Vanessa Checchetto, Sara De Bortoli, Elide Formentin, Alex Costa, Ildikó Szabó, Enrico Teardo

**Affiliations:** ^1^Department of Biology, University of PadovaPadova, Italy; ^2^Department of Biomedical Sciences, University of PadovaPadova, Italy; ^3^Department of Life Science and Biotechnology, University of FerraraFerrara, Italy; ^4^Department of Biosciences, University of MilanMilan, Italy; ^5^CNR, Institute of Biophysics, Consiglio Nazionale delle RicercheMilan, Italy; ^6^CNR, Institute of NeurosciencesPadova, Italy

**Keywords:** higher plants, mitochondria, calcium channels and transporters, calcium homeostasis, physiological processes

## Abstract

Plants, being sessile organisms, have evolved the ability to integrate external stimuli into metabolic and developmental signals. A wide variety of signals, including abiotic, biotic, and developmental stimuli, were observed to evoke specific spatio-temporal Ca^2+^ transients which are further transduced by Ca^2+^ sensor proteins into a transcriptional and metabolic response. Most of the research on Ca^2+^ signaling in plants has been focused on the transport mechanisms for Ca^2+^ across the plasma- and the vacuolar membranes as well as on the components involved in decoding of cytoplasmic Ca^2+^ signals, but how intracellular organelles such as mitochondria are involved in the process of Ca^2+^ signaling is just emerging. The combination of the molecular players and the elicitors of Ca^2+^ signaling in mitochondria together with newly generated detection systems for measuring organellar Ca^2+^ concentrations in plants has started to provide fruitful grounds for further discoveries. In the present review we give an updated overview of the currently identified/hypothesized pathways, such as voltage-dependent anion channels, homologs of the mammalian mitochondrial uniporter (MCU), LETM1, a plant glutamate receptor family member, adenine nucleotide/phosphate carriers and the permeability transition pore (PTP), that may contribute to the transport of Ca^2+^ across the outer and inner mitochondrial membranes in plants. We briefly discuss the relevance of the mitochondrial Ca^2+^ homeostasis for ensuring optimal bioenergetic performance of this organelle.

## Introduction

### Mitochondria and Calcium Homeostasis

**FIGURE 1:**
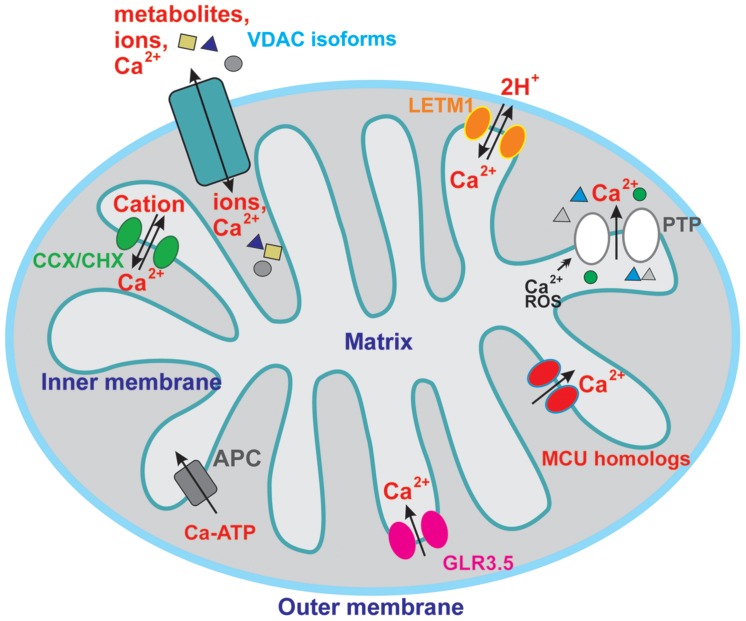
**Overview of the putative calcium-permeable ion channels and transporters in plant mitochondria.** For sake of clarity, only channels and transporters putatively involved in calcium transport are shown, so the channels mediating flux of other ions are not depicted here. See text for further details.

Molecular identification and pharmacological characterization of mitochondria-located ion channels allowed a deep understanding of the crucial importance of these proteins for organelle function and even for determining cell fate in animals (Leanza et al., 2014; Szabo and Zoratti, 2014). In plant mitochondria the current knowledge is unfortunately more limited than in the animal system and only few electrophysiological studies deal with plant mitochondrial ion channels. These include the voltage-dependent anion channel (VDAC), e.g., (Abrecht et al., 2000; Berrier et al., 2015) of the outer mitochondrial membrane (OMM) and those of the inner membrane (IMM), i.e., a large conductance Ca^2+^-insensitive potassium channel (Matkovic et al., 2011), a mitochondrial chloride channel (Matkovic et al., 2011) presumably corresponding to PIMAC (plant inner membrane anion channel) studied by classical bioenergetics (for review see Laus et al., 2008), a large-conductance Ca^2+^-activated BK-type potassium channel (Koszela-Piotrowska et al., 2009) and an ATP-dependent potassium channel K (ATP; De Marchi et al., 2010; Jarmuszkiewicz et al., 2010; Matkovic et al., 2011). In almost all studies either purified inner mitochondrial vesicles or purified proteins have been exploited upon incorporation into artificial membrane via the black lipid bilayer technique, because application of the patch clamp electrophysiological technique is experimentally very demanding (De Marchi et al., 2010). In addition to electrophysiological investigation, in-depth bioenergetic studies proved the existence and the relevance of potassium-permeable pathways (e.g., Pastore et al., 1999; Trono et al., 2014, 2015), of anion transport (Laus et al., 2008), of the proton-gradient dissipating uncoupling proteins (Vercesi et al., 2006) and of the permeability transition pore (Vianello et al., 2012; Zancani et al., 2015) in the context of plant mitochondrial physiology. Plant mitochondria have been shown to contain an uptake system for Ca^2+^ as well (Hanson et al., 1965; Dieter and Marme, 1980; Martins and Vercesi, 1985; Carnieri et al., 1987; Silva et al., 1992; Zottini and Zannoni, 1993): however, the molecular identification of the pathways mediating Ca^2+^ flux is still far from complete. **Figure 1** summarizes the currently known/hypothesized pathways in plant mitochondria.

Understanding the routes and regulation of mitochondrial Ca^2+^ entry and exit would bring to an important advancement in the field and would help to determine the impact of Ca^2+^ homeostasis on bioenergetic efficiency and on the function of this organelle in plants. It has to be underlined that amongst the different signal transduction mechanisms Ca^2+^ plays a prominent role as a secondary messenger. A highly negative membrane potential in mitochondria (-150 to -180 mV) represents a huge driving force for the uptake of Ca^2+^ and other cations (Szabo and Zoratti, 2014) therefore calcium influx and efflux have to be under tight control in order to avoid calcium overload. In animal cells, transient accumulation of Ca^2+^ in intracellular organelles shapes cytosolic Ca^2+^ signals (Rizzuto et al., 2012) and a similar concept has been suggested for plants (Stael et al., 2012; Nomura and Shiina, 2014). The emerging view is that the influx of Ca^2+^ into the mitochondrial matrix can be induced by a variety of abiotic stresses such as heat, oxidative and salt stress, anoxia or extracellular ATP and extracellular Ca^2+^ (Logan and Knight, 2003; Xiong et al., 2006; Loro et al., 2012; Schwarzlander et al., 2012; Zhao et al., 2013; Rikhvanov et al., 2014; He et al., 2015; Pu et al., 2015), but the extent of uptake is variable among different tissues and species (Martins and Vercesi, 1985) as is the case also for the animals, (e.g., Fieni et al., 2012). The so far available data suggest that Ca^2+^ fluxes into and out of mitochondria might also shape the cytosolic “Ca^2+^ signature” (Stael et al., 2012; Nomura and Shiina, 2014), however, direct *in vivo* experimental proof has to be obtained in favor of this idea. In fact, in a recent study, where the calcium uptake into mitochondria has been impaired by deletion of the MCU regulator MICU (see below), cytoplasmic calcium transients appeared unaltered (Wagner et al., 2015).

Clear-cut demonstration of stimuli-induced Ca^2+^ uptake into mitochondria in intact plant tissues has been obtained thanks to the mitochondria-targeted genetically encoded Ca^2+^ sensors, aequorin (Logan and Knight, 2003) and Cameleon (Loro et al., 2012). Mitochondrial Ca^2+^ accumulation correlated to the intensity of Ca^2+^ increase in the cytoplasm. However, the molecular players of Ca^2+^ uptake and release remain to be largely elucidated. In addition, mostly indirect evidence indicate that in plant mitochondria, similarly to animal ones (Rizzuto et al., 2012), matrix Ca^2+^ levels regulate tricarboxylic acid (TCA) cycle enzymes (He et al., 2015) and oxidative phosphorylation (Wagner et al., 2015).

### Ca^2+^-Flux Mediating Pathways in Animal Mitochondrial Membranes

Several players participating in calcium homeostasis have been identified over the last decades. We briefly mention these pathways, although it has to be kept in mind, that while knowledge from the animal field might be a source of inspiration, plant mitochondria do not necessarily take advantage of the same systems.

Voltage-dependent anion channel (VDAC) isoforms are universally recognized as the main pathways for flux of metabolites and ions across the OMM (see, e.g., Shoshan-Barmatz et al., 2010; Checchetto et al., 2014; Mertins et al., 2014; Madamba et al., 2015). VDACs, although defined as anion channels, can conduct a substantial flow of Ca^2+^, as demonstrated both *in vitro* and *in vivo* for the mammalian protein (Gincel et al., 2001; Rapizzi et al., 2002; Bathori et al., 2006; Israelson et al., 2007; Rizzuto et al., 2009; Shoshan-Barmatz et al., 2010). As to Ca^2+^ uptake across the IMM, the 40 kDa protein (MCU) has been proposed to be the channel-forming component of the Ca^2+^ uniporter (Baughman, 2011; De Stefani et al., 2011). The uptake of Ca^2+^ via the uniporter has been linked to energy production (De Stefani et al., 2015). Lack of MCU in knock-out mice causes only modest defects in skeletal muscle strength and minor metabolic changes (Pan et al., 2013; Pendin et al., 2014) but MCU was shown to be important for bioenergetic performance in other models (Huang et al., 2013; Wu et al., 2015). In addition to MCU, several additional proteins such as MICU1 (Perocchi et al., 2010), EMRE (Sancak et al., 2013), MCUR1 (Mallilankaraman et al., 2012), and MCUb (a dominant negative MCU isoform; Raffaello et al., 2013) as well as MICU2 (Patron et al., 2014) were reported to be essential components and/or regulators of the mammalian MCU complex (MCUC; De Stefani et al., 2015; Foskett and Philipson, 2015). MICU1/2 proteins are EF-hand proteins with the ability to regulate mitochondrial Ca^2+^ uptake and MCU channel activity upon Ca^2+^ binding (Patron et al., 2014). EMRE was proposed to mediate the physical interaction between MCU and MICU1/MICU2 dimer and has recently been shown to fine-tune calcium-regulation of the channel activity on the matrix side (Vais et al., 2016). MCUR1 also affects mitochondrial Ca^2+^ uptake, however the underlying mechanism is still highly debated (Paupe et al., 2015; Vais et al., 2015).

Mitochondrial Ca^2+^ uptake might take place via additional or alternative components as well. In animals, LETM1 has been proposed to possess a Ca^2+^/2H^+^ electroneutral antiporter activity and to take up Ca^2+^ (Jiang et al., 2013; Doonan et al., 2014; Tsai et al., 2014). The yeast homolog Mdm38 however affects potassium homeostasis (Nowikovsky et al., 2004) and impacts mitochondrial translation, independently of its ion transporter function (Bauerschmitt et al., 2010). The nature of the ions transported by LETM1 is still highly debated (Nowikovsky et al., 2012; Nowikovsky and Bernardi, 2014). In addition, the solute carrier 25A23 (SLC25A23) has been shown to interact with MCU and was proposed to play an important role in mitochondrial Ca^2+^ influx (Hoffman et al., 2014).

It also has to be mentioned that calcium uptake is linked to cell death pathways via induction of the PTP (Bernardi et al., 2015). The PTP, first characterized in mammalian cells, is a channel responsible for the permeability increase of the inner mitochondrial membrane under specific conditions (Zoratti and Szabo, 1995; Bernardi et al., 2015). PTP can be activated by different stimuli such as high matrix Ca^2+^ concentration (in 100s μM range) and oxidative stress, leading to swelling of mitochondria and dissipation of energy. In animals, when PTP opens only transiently, it possibly mediates Ca^2+^ release from mitochondria (Bernardi and von Stockum, 2012) together with the recently identified 3Na^+^/Ca^2+^ antiporter (NCX; Palty et al., 2010). In addition, a still unidentified Na^+^-insensitive Ca^2+^ release system, possibly a H^+^/Ca^2+^ antiporter plays a role (Nowikovsky et al., 2012). Over the last decades VDAC, the adenine nucleotide carrier, the benzodiazepine receptor and cyclophilin D (CypD) were proposed in different combinations as the main components of the mammalian PTP (Zoratti and Szabo, 1995; Bernardi et al., 2015). The discovery that oxidative stress and application of elevated [Ca^2+^] results in channel formation by the dimeric form of the F-ATP synthase, opened a new perspective to the field (Giorgio et al., 2013). A recent work proposed instead, that mitochondrial spastic paraplegia 7 (SPG7), a nuclear-encoded mitochondrial metalloprotease (m-AAA) which interacts with CypD and VDAC1 and with a paraplegin-like protein AFG3L2, is essential for the PTP complex formation (Shanmughapriya et al., 2015). However, PTP could still be opened in the absence of SPG7, although at higher matrix calcium concentrations, suggesting that SPG7, similarly to CypD, acts a regulator rather than a crucial pore-forming moiety of the PTP (Bernardi and Forte, 2015). Interestingly, apart from their proteolytic roles, the *m*-AAA proteases mediate ATP-dependent membrane dislocation of the heme-binding reactive oxygen scavenger protein Ccp1 (Tatsuta et al., 2007), possibly linking PTP activation to oxidative stress.

## Ca^2+^ Flux-Mediating Pathways in Plant Mitochondrial Membranes

### VDAC of the Outer Mitochondrial Membrane

In higher plants, similarly to animals, functionally distinct isoforms of VDAC exist. In particular, in *Arabidopsis* various isoforms displaying distinct subcellular localization (in the plasma membrane, mitochondria, chloroplasts, and plastids) and function have been identified (Smack and Colombini, 1985; Pottosin, 1993; Clausen et al., 2004; Tateda et al., 2011; Homble et al., 2012; Robert et al., 2012; Takahashi and Tateda, 2013; Michaud et al., 2014). Their roles, as assessed mostly by using T-DNA insertion knockout mutants of *Arabidopsis*, include regulation of development (Tateda et al., 2011; Robert et al., 2012; Pan et al., 2014), regulation of the hypersensitive response/programmed cell death (Lacomme and Roby, 1999; Tateda et al., 2009, 2011), of the response to abiotic stress (Li et al., 2013; Zhang et al., 2015) and import of tRNA into mitochondria (Salinas et al., 2006). Whether any of these functions requires Ca^2+^ flux across the OMM (or across other membranes) mediated by VDACs is unclear, however AtVDAC1 has been shown to interact in two-hybrid yeast system with CBL1, a Ca^2+^-sensor (Li et al., 2013). Despite detailed electrophysiological characterization of several isoforms (Blumenthal et al., 1993; Pottosin, 1993; Mlayeh et al., 2010; Godbole et al., 2011; Berrier et al., 2015), experimental evidence is still missing to understand whether and how VDAC proteins influence Ca^2+^ flux across plant endomembranes.

### Homologs of the Mitochondrial Calcium Uniporter (MCU) in the Inner Mitochondrial Membrane

In the *Arabidopsis thaliana* genome six genes are present which can be identified as putative MCU channel proteins with predicted mitochondrial targeting, since they display sequence similarity with the mammalian MCU counterparts and contain the conserved DVME (Asp-Val-Met-Glu) selectivity filter sequence (Stael et al., 2012). Varying number of homologs can be identified in the genome of other higher plants as well (see Aramemnon http://aramemnon.uni-koeln.de/). Whether all these isoforms are indeed targeted to mitochondria, whether they form ion channels able to provide a permeation pathway for Ca^2+^ and whether the various isoforms operate in different tissues and/or at different developmental stages still awaits clarification. Recently obtained experimental evidence indicates that at least one of the isoforms is indeed targeted to mitochondria in *Arabidopsis* (Carraretto et al., 2016). The discovery showing that lack of the only existing isoform of the regulator, AtMICU, in *Arabidopsis* alters mitochondrial Ca^2+^ uptake points to a functional conservation of the core-components of the MCU complex in plants (Wagner et al., 2015). Interestingly, even though Ca^2+^ uptake into mitochondria and basal Ca^2+^ are significantly higher in AtMICU-less plants than in WT plants, respiration and mitochondrial morphology are only slightly affected and plant development is normal (Wagner et al., 2015). As to EMRE, its close homologs do not seem to be present in higher plants, while one of the two homologs of MCUR1 in *Arabidopsis* has been described as a plant specific subunit of complex IV (Millar et al., 2004; Klodmann et al., 2011). In summary, the plant homologs of MCU and MICU1 are certainly excellent candidates to be key players in mitochondrial Ca^2+^ homeostasis, but experimental proof for the ability of MCU proteins to form Ca^2+^-permeable channels is still lacking.

### Glutamate Receptor 3.5, LETM1/Mdm38 and Adenine Nucleotide/Phosphate Carriers (APCs) of the Inner Membrane

Similarly to the animal mitochondria, alternative calcium flux-mediating pathways seem to exist also in higher plants. Our knowledge in this respect is restricted mostly to *Arabidopsis*, since T-DNA insertion mutants of this model plant are available and widely used. In *Arabidopsis* deletion of both isoforms of LETM is lethal, probably due to the requirement of LETM proteins for mitochondrial protein translation and accumulation (Zhang et al., 2012). A recent study provided evidence that members of the APC family of *Arabidopsis* mediate a time dependent uptake of [^45^Ca] (in the form of Ca-ATP) *in vitro* (Lorenz et al., 2015). This system is homolog of the above mentioned SLC25A23. The transport rate of AtAPC2 was however low and was completely blocked by 25-fold excess of Mg^2+^, suggesting that *in vivo* Ca^2+^ flux might take place via this transporter only under specific conditions in plant mitochondria.

Another study located an alternative spliced isoform of a member of the glutamate receptor family, AtGLR3.5 to mitochondria. Although there is no direct evidence thus far demonstrating that the subfamily 3 member AtGLR3.5 functions as Ca^2+^-permeable ion channel, the close homolog AtGLR3.4 and the AtGLR1.4 and AtGLR1.1 pores behave as Ca^2+^-permeable non-selective cation channels when expressed in heterologous systems (Tapken and Hollmann, 2008; Vincill et al., 2012; Tapken et al., 2013). In addition, inner membrane vesicles isolated from spinach chloroplasts and containing members of the GLR subfamily 3 harbor a glutamate/glycine-induced Ca^2+^-permeable activity which is sensitive to known animal ionotropic glutamate receptor antagonists (Teardo et al., 2010). Studies using *Atglr3.3* mutant plants showed that Ca^2+^ uptake induced by glutamate in *Arabidopsis* into hypocotyls and root cells is correlated with the presence of AtGLR3.3 (Qi et al., 2006). Therefore, similarly to the other members of the subfamily 3, AtGLR3.5 is expected to work as Ca^2+^-permeable channel. In accordance, Ca^2+^ dynamics measurements performed using the Cameleon probe targeted to mitochondria in WT and mutant plants lacking AtGLR3.5 revealed that AtGLR3.5 might mediate Ca^2+^ uptake into mitochondria, at least in response to specific stimuli (e.g., wounding (Teardo et al., 2015).

In agreement with the mitochondrial localization and predicted activity of AtGLR3.5, plants lacking this protein harbor mitochondria with profoundly altered ultrastructure: a dramatic loss of cristae and swelling with the matrix becoming translucent can be observed. The exact mechanism leading to these changes is not known, even though *AtGLR3.5* topology indicates that the glutamate (agonist)-binding domains are located in the cytosol, possibly sensing the cytosolic glutamate/aminoacid concentration (Teardo et al., 2015). Whether, a reduced Ca^2+^ uptake in the *Atglr3.5* KO plants leads to morphological changes via reduction of the oxidative phosphorylation remains to be established. It is interesting to note that complex I inhibition in mammalian mitochondria leads to similar morphological changes to that observed for *Atglr3.5* KO plants (Ramonet et al., 2013).

### Permeability Transition Pore

Plant mitochondria can also undergo Ca^2+^-induced permeability transition, (Arpagaus et al., 2002; Petrussa et al., 2004; Vianello et al., 2012), an event linked to nitric oxide-induced cell death (Saviani et al., 2002) and to programmed cell death (for updated review see Zancani et al., 2015). Whether the “life-sustaining” F-ATP synthase forms the PTP in plants as well, still has to be clarified, but the thylakoid membrane, which also contains this ATP-producing machinery, harbors a high-conductance channel resembling PTP (Hinnah and Wagner, 1998). Ccp1 (see above) shares high sequence homology with ascorbate peroxidase (APX), with one of the isoforms dually targeted to mitochondria and chloroplasts (Chew et al., 2003). This further supports previous findings in the literature that ROS regulates PTP also in plants. A BLAST search in the *Arabidopsis* database reveals that it contains several ATP-dependent metalloproteases (FtsH proteases) that show high aminoacid sequence similarity to SPG7 (see above; e-values ranging from 2e^-169^ to 8e^-58^, with aminoacid identities in the range of 36–49%) and to AFG3L2 (e-value of 0 and 50% identity is found for AtFtsH3 and AtFtsH10). Among those with the highest score, AtFtsH3 and AtFtsH10 are present in mitochondria (Piechota et al., 2010).

Several plant putative Ca^2+^/cation exchangers, for example CCX1, CCX3, and CCX4 (Schwacke et al., 2003), score for mitochondrial localization according to Aramemnon database although in most species rather poorly. Experimental evidence in favor of their localization in mitochondria versus secretory pathways or of a dual localization is missing. Furthermore, for AtCCX3 it has been established that it functions as an endomembrane-localized H^+^-dependent K^+^ transporter (Morris et al., 2008). A putative cation/proton exchanger, AtCHX25 has also a predicted mitochondrial localization, but whether it mediates K^+^/H^+^ exchange like some members of the AtCHX family or Ca^2+^/H^+^ exchange has still to be established (Sze et al., 2004; Evans et al., 2012; Chanroj et al., 2013).

Under biologically relevant conditions (e.g., during oxidative stress), the above-described uptake and efflux pathways for calcium might cooperate. For example, Ca^2+^ influx-triggered Ca^2+^ release has been linked to pulsing of the mitochondrial membrane potential, a phenomena proposed to yield a transient uncoupling leading to reduced ROS production (Schwarzlander et al., 2012).

In summary, a combination of genetics, fluorescent probe imaging, electrophysiology, bioenergetics and physiology will hopefully provide answers to the numerous open questions related to mitochondrial calcium homeostasis in plants.

## Author Contributions

All authors contributed to the works published by our groups which are described in the minireview. Furthermore, all authors actively participated in writing the manuscript.

## Conflict of Interest Statement

The authors declare that the research was conducted in the absence of any commercial or financial relationships that could be construed as a potential conflict of interest.
